# Aptamer-based applications for cardiovascular disease

**DOI:** 10.3389/fbioe.2022.1002285

**Published:** 2022-10-13

**Authors:** Xinyuan Chen, Yue Ma, Yuquan Xie, Jun Pu

**Affiliations:** Department of Cardiology, Renji Hospital, School of Medicine, Shanghai Jiao Tong University, Shanghai, China

**Keywords:** aptamer, precision medicine, cardiovascular disease, atherosclerosis, von Willebrand factor, antithrombotic therapy

## Abstract

Cardiovascular disease (especially atherosclerosis) is a major cause of death worldwide, and novel diagnostic tools and treatments for this disease are urgently needed. Aptamers are single-stranded oligonucleotides that specifically recognize and bind to the targets by forming unique structures *in vivo*, enabling them to rival antibodies in cardiac applications. Chemically synthesized aptamers can be readily modified in a site-specific way, so they have been engineered in the diagnosis of cardiac diseases and anti-thrombosis therapeutics. Von Willebrand Factor plays a unique role in the formation of thrombus, and as an aptamer targeting molecule, has shown initial success in antithrombotic treatment. A combination of von Willebrand Factor and nucleic acid aptamers can effectively inhibit the progression of blood clots, presenting a positive diagnosis and therapeutic effect, as well as laying a novel theory and strategy to improve biocompatibility paclitaxel drug balloon or implanted stent in the future. This review summarizes aptamer-based applications in cardiovascular disease, including biomarker discovery and future management strategy. Although relevant applications are relatively new, the significant advancements achieved have demonstrated that aptamers can be promising agents to realize the integration of diagnosis and therapy in cardiac research.

## Introduction

**FIGURE 1:**
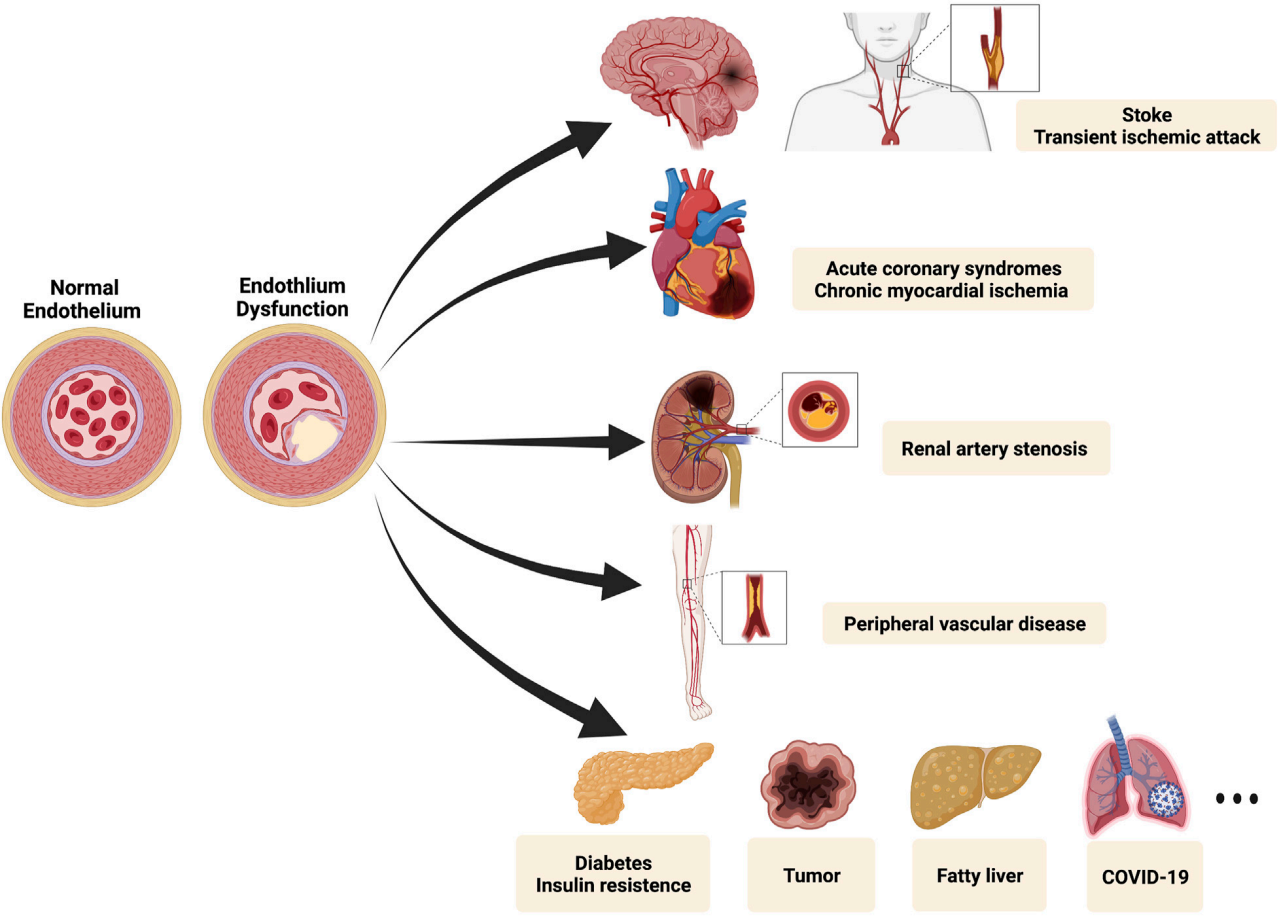
The spectrum of the panvascular system. Endothelial dysfunction is considered as a hallmark of diverse panvascular diseases. Major lesioned vessels involve carotid arteries, cerebral arteries, coronary arteries, renal arteries, and peripheral vessels. Besides relevant clinical manifestations include stroke, transient ischemic attack, acute coronary syndromes (myocardial infarction and unstable angina), chronic myocardial ischemia (stable angina and silent ischemia), renal artery stenosis, peripheral vascular diseases (limb ischemia and intermittent claudication), metabolic syndrome (diabetes and insulin resistance), and many others (tumor, fatty liver, COVID-19, *etc.*). Created with BioRender.com.

Cardiovascular disease (CVD) is one of the major causes of death globally. Of CVD-related deaths, 45.1% are accounted by coronary artery diseases (CAD), defined as a decrease or interruption of blood flow due to atherosclerotic plaque occlusion (Niehoff et al., 2022; Bejarano et al., 2018). With the advancement of medical research, scholars recognized the limitations of studying the mechanism of diseases separately and commence to focus on the pathological process of vascular disorders systematically. The concept of panvascular disease was firstly introduced by Lanzer and Topol in 2002, based on a unified understanding of vascular systemic diseases (Lanzer and Topol, 2002). Panvascular refers to a complex network of human vasculature, composed of arteries, veins, and lymphatic vessels, which is the “irrigation channel” of vital organs and the “lifeline” of human health. As a group of systemic vascular disorders, panvascular diseases share common pathological features (most of which are atherosclerosis), mainly undermining major organs such as the heart, brain, kidney, extremities, and aorta (Herrington et al., 2016). As shown in Figure 1, atherosclerosis is characterized by the formation of lipid lesions within the artery trees that involve multiple vessels (coronary artery, cerebral arteria, peripheral vessels, *etc.*), ultimately leading to myocardial infarction (MI), strokes, and peripheral vascular diseases (Kobiyama and Ley, 2018). Several factors affect atherosclerosis, most of which include high blood pressure, hyperlipidemia, cigarette smoking, disturbed sleep, and environmental stress (Libby, 2021). Because of atherosclerosis plaques’ slow progression, most patients remain asymptomatic for years. When symptoms do occur, they usually relate to plaque ruptures and thrombosis, leading to unbalance between the demand and supply of oxygen (Libby et al., 2019). As early detection of atherosclerosis remains elusive, achieving portable, cost-effective, high-accurate devices interrogating cardiovascular diseases is inevitable.

Aptamers, special nucleic acid ligands, serve as novel recognition tools with high affinity and specificity to their targets. They are short single-stranded oligonucleotides (12–80 nt) that fold into unique tertiary structures, enabling them to tightly bind with cognate targets ranging from biomolecules to circulating cells, exosomes, microRNA (Zhu and Chen, 2018; Wu et al., 2021). Aptamers can be evolved by an *in vitro* iterative process known as Systematic Evolution of Ligands by Exponential Enrichment (SELEX), firstly described in 1990 by Tuerk and Gold and Ellington and Szostak (Ellington and Szostak, 1990; Tuerk and Gold, 1990), which uses a series of target binding, washing, and amplification steps to select single-stranded (ss) DNA or RNA based on the principle of Watson-Crick base pairing (Mittal et al., 2018). As technologies of recombinant and engineering methodologies developed rapidly, aptamers can be easily generated and adjusted *via* sequence modifications *in vitro*, as well as synthesized in a large quantity to reduce batch-to-batch variations. Compared with other classes of ligands such as antibodies or peptides, aptamers also have several distinctive advantages, including lower toxicities and immunogenicity. Besides, aptamers are stable over high temperatures and easy to transport (Zhong et al., 2020). Also, oligonucleotides containing sequences complementary to the functionalized aptamers can efficiently neutralize the effects of these moieties (Zhao et al., 2021). Given the characteristic merits of aptamers, they have received extensive attention in the biomedical field and have been widely applied in many fields, such as environmental monitoring, material science, and drug screening (Huang et al., 2021). In this review, we provide an overview of several contemporary preclinical or clinical aptamer applications in cardiac diseases, especially in biomarker detection, cardiovascular disease diagnosis, and therapy. Finally, we discuss the potential challenges and future perspectives in the aptamer field.

## Aptamer-based cardiac disease diagnosis

### Aptamers in cardiac biomarker discovery

In the 1980s, the introduction of immunochemical assays lifted the curtains of plasma biomarkers. Currently, the plasma proteome, reflecting the states or phenotype of the individuals, has been central to clinical decision-making (Smith and Gerszten, 2017). The plasma proteome can be classified into three classes: functional proteins (human serum albumin, apolipoproteins), tissue leakage proteins (creatine kinase, cardiac troponins), and signal proteins (hormones, cytokines). However, the low abundance of target proteins and restricted sensitivity of detection techniques hinder the accuracy of diagnosis of cardiovascular disease (Geyer et al., 2017; Smith and Gerszten, 2017). We urgently need to introduce new methods to improve the key capabilities of multiplexing, high sample throughput, robustness, the sensitivity for clinical protein biomarkers in cardiac diseases.

Several proteomic technologies for biomarker discovery have been reviewed (Smith and Gerszten, 2017; Joshi et al., 2021). Electrophoresis and chromatography are the earliest implements for protein discovery, based on the characteristics such as charge, mass, and mobility, while these tools suffer from poor resolution and are limited to detecting the most abundant component of a liquid mixture (Smith and Gerszten, 2017). Mass spectrometry (MS) measures fragmentation patterns of peptides derived from the digestion of proteins. Due to the unique sequence of these peptides, MS and related analytical tools are shown to be unbiased and precise (Geyer et al., 2017; Smith and Gerszten, 2017). However, the complicated process of multiple sample preparation hinders the utilization in reliably identifying and quantifying biomolecules of human clinical plasma samples, as well as the lack of reproducible and high-throughput workflows (Gerszten et al., 2008). An alternative strategy to address the restrictions of proteomics discovery has been the development of affinity reagents, notably the aptamer which can be easily detected by Polymerase Chain Reaction (PCR) or hybridization tools (Smith and Gerszten, 2017).

**FIGURE 2:**
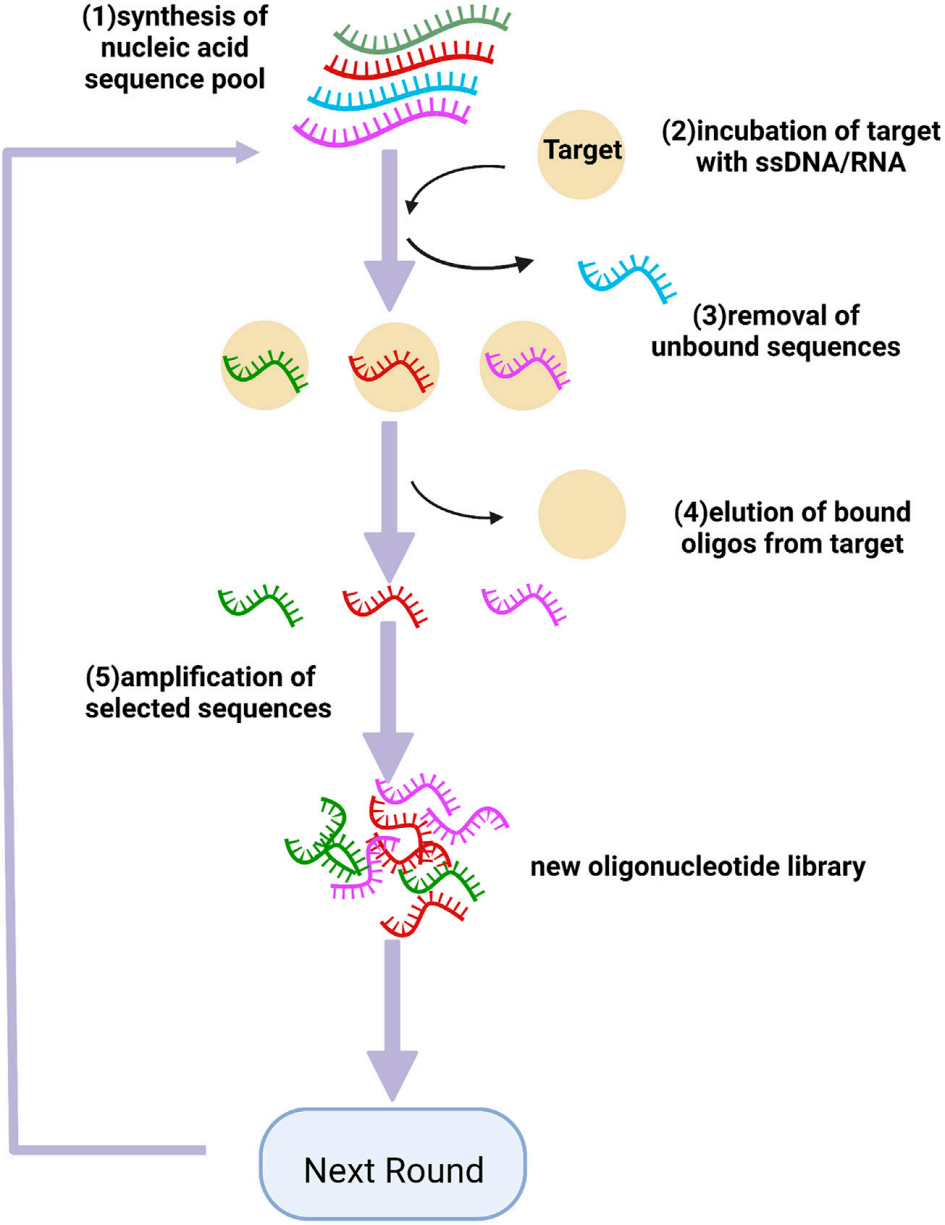
Schematics of SELEX for aptamer selection. (1) synthesis of the nucleic acid pool which contained a central random sequence containing 20–50 bases; (2) incubation of target with the library; (3) removing the unbound oligonucleotides from those bound to the target molecule; (4) eluting the bound oligos from the target; (5) amplifying the selected oligos to generate a new pool for the next ground. Created with BioRender.com.

SELEX is a powerful modality that can further biomarker discovery, firstly reported in 1990 (Tuerk and Gold, 1990), including magnetic bead-based SELEX (Shatila et al., 2020), capillary electrophoresis-SELEX (CE-SELEX) (Zhu et al., 2021), and sol-gel SELEX (Bae et al., 2013). Each protein has its detection agent in DNA/RNA sequence libraries. As shown in Figure 2, multiple steps were involved in the SELEX process, beginning with nucleic acid pool synthesis. Then target is incubated with a mixture of modified oligos to produce target-oligos complexes. Next, bound ssDNA/RNA are separated, selected, eluted, cloned, and sequenced (Cen et al., 2021). In order to improve the efficiency of aptamer discovery procedures, efficient selection platforms are adopted to SELEX process, involving nitrocellulose filtration and microfluidics. In order to improve the pharmacological properties of aptamers, various chemical modifications have been performed, stabilizing them against nuclease degradation (Kovacevic et al., 2018). These modification sites involve phosphodiester linkage, sugar ring, and a terminal end, allowing aptamers to incorporate with target molecules with higher affinity (Koudrina et al., 2020). The small size of aptamers makes them promptly leak from the kidney and the active duration is not too long, so how to extend their retention time remains the main issue. Almost every aptamer has some modification to be more thermostable *in vivo*, and one of the major strategies is pegylation (Kovacevic et al., 2018). Aptamers pegylated with polyethylene glycols (PEG) significantly increase the weight of the molecules to evade the glomerular filtration, to extend the circulating time, yet some studies exposed that this modification may impede the affinity of the nucleotides, and lead to hypersensitivity reaction induced by PEG (Lin et al., 2021). Recently, a new strategy assembling gold nanoclusters (GNCs) with aptamers *via* self-electronic interactions, has been reported and shown simple engineering process and longer residence time, as well as higher affinity, verified by Sgc8 in cancer imaging test (Xia et al., 2021), avoiding the possible immunogenicity of large molecules. Although so far, a lot of researches dedicated to prolonging the residence time of aptamers *in vivo*, their pharmacokinetic property remains nonstable and further optimization is still needed. Furthermore, the modifications on the aptamers also includes adding probes, such as fluorochromes, to detect target proteins. The procedures of how aptamer quantified plasma proteins have been previously described (Chan et al., 2020; Ferrannini et al., 2020). SOMAscan assay (SomaLogic, Inc.), a high-performance proteomic profiling platform, is based on the Slow Off-rate Modified Aptamer (SOMAmer), firstly introduced by professor Gold, which contains pyrimidine residues modified with amino acid side chain mimics at 5’ ends and are qualified with slow dissociation kinetics (t_1/2_ > 30min), achieving innovations of high-throughput and unbiased proteomics study (Gold et al., 2010). In recent research, Ferrannini and co-workers analyzed plasma proteins by aptamer assays and reported that GDF15, renin, and adiponectin may be shared markers of type 2 diabetes (T2D) and CAD. Besides, lower plasma renin levels in patients with T2D may protect them against CAD (Ferrannini et al., 2020). In a cross-sectional study, qualification (by aptamer technology), ranking (using partial least squares), and correlations (by multivariate regression) were performed and four proteins have been found that are associated with opposed vascular processes (healing vs adverse remodeling), two of four proteins showing a protective role against CAD, myosin regulatory light chain 2 atrial isoform (MYO or MLC-2a) and C-C motif chemokine 22 (C-C 22). The other two proteins, protein Shisa 3 homolog (PS-3) and platelet-activating factor acetylhydrolase (PAF-AH), may be related to high CAD burden in low-risk subjects (Ferrannini et al., 2021). Chan et al. used affinity-based aptamer probes, cross-referenced to single-cell transcriptomic analysis to explore protein candidates related to post-MI heart failure (HF), and identified 83 proteins as potential biomarkers, of which 4 highest-priority are nascent (angiopoietin-2, thrombospondin-2, latent transforming growth factor beta binding protein 4, and follistatin-related protein 3) Chan et al. (2020). Moreover, two large community-based cohorts (Framingham Heart Study and HUNT study) using the SOMAscan platform also identified new circulating biomarkers of HF, three proteins (thrombospondin-2, mannose-binding lectin, N-terminal proB-type natriuretic peptide) related to higher HF risk, while three (epidermal growth factor receptor, growth differentiation factor-11/8, hemojuvelin) showing cardiac protection (Nayor et al., 2020). So far, many novel proteins have been identified by highly-multiplex aptamer-based proteomic platforms, which are associated with cardiac metabolism, mechanosensation, and vascular homeostasis, involving zeta chain of T cell receptor-associated protein kinase 70, aminoacylase-1, apolipoprotein M, adenylosuccinate synthase-like protein 1, *etc.* (Benson et al., 2018; Jacob et al., 2018; Chirinos et al., 2020; Katz et al., 2022). Currently, the utility of left ventricular ejection fraction (LVEF) is the most reliable clinical determinant of identifying functional and structural phenotyping in HF. Patients can be stratified within three LVEF classifications: HF with reduced (HFrEF, LVEF <40%), mid-range (HFmrEF, LVEF 40%–50%), and preserved (HFpEF, LVEF >50%) ejection fraction (Bristow et al., 2017; Adamo et al., 2020). Adamo et al. using aptamer technology, have found that patients with different HF categories had distinct variations in plasma proteomics, arguing that a combination of LVEF with multiplexed proteomics assays may be optimal to assess clinical phenotypes of patients with HF, as well as predict the prognosis of HF patients after medical management (Adamo et al., 2020). These findings may contribute to a more in-depth understanding of the heart disease process beyond traditional risk status assessment and potentially constitute new targets for treatment.

### Aptamer-based cardiovascular diagnosis

Identification of biomarkers will facilitate cardiovascular risk assessment, diagnosis and prognosis, because they can reflect individuals’ physiological conditions dynamically The concept of aptasensor was first introduced in the early 2000s (Stojanovic et al., 2001). Coupled with various probes and corresponding detection technologies (fluorescence imaging, electrochemical technique, and chemiluminescence immunoassay), aptamers can be used in disease diagnosis (Ma et al., 2015). Currently, biomarker measurement is one of the most important laboratory tests for the diagnosis of cardiovascular disease (Negahdary, 2020).

Cardiac troponin I (cTnI), a biomarker of myocardial injury, is recommended by international guidelines as the gold standard for diagnosis of MI and is mainly measured by high-sensitivity (hs-cTnI) kits prepared with antibodies (Clerico et al., 2020; Cen et al., 2021). Cen et al. screened aptamers (96 bp), specifically bound to cTnI, by magnetic bead-SELEX technology and finally selected two optimal aptamers (Apt3, Apt6) to develop dual-aptamer (Apt3-Apt6) sandwich ELONA assays, expanding the detection range at 0.05–200 ng/ml, and also improving the detection accuracy Cen et al. (2021). In recent research, an ultrasensitive electrochemical biosensor that combined TdT signal amplification strategy with cTnI aptamers has been developed, achieving the detection level of cTnI at 40 pg/ml. Nanotechnology shows its nanometric effects on biosensors, such as enhanced surface area, high reactivity, and special physicochemical properties. Up to now, researchers have designed a series of nanostructure-based aptasensors, and various nanostructures, such as gold, carbon, and silica (Wang et al., 2017) have been used in the engineering of biosensors. In the latest study, molybdenum disulfide (MoS_2_) nanosheets were developed through a tuned hydrothermal method and coated on the surface of the SPE electrode, then cTnI aptamer was immobilized with it. This mentioned aptasensor can detect cTnI at a concentration as low as 10 fM, with a high degree of selectivity (Vasudevan et al., 2021). In another aptasensor, nanodiamonds (NDs) and hydrogen-substituted graphdiyne (HsGDY) were crossed together as a nanohybrid and applied as an anchor to secure aptamer strands for detecting biomarkers, such as myoglobin (Myo) and cTnI, giving the limit of detection (LOD) of 6.29 and 9.04 fg/ml respectively (Wang et al., 2021). Besides nanostructure aptamers combined with the clustered regularly interspaced short palindromic repeat (CRISPR), a powerful tool for genome engineering further improved the sensitivity of cTnI detection. A gold electrode was considered as the working electrode and decorated with the methylene blue-modified DNA (probe1). Then cTnI aptamer with modified magnetic nanoparticles was binding to its complement DNA (probe2) in part. When the aptamer captured the cTnI, probe2 was released and hybridized with the CRISPR-derived RNA in the solution and thereby triggered the trans-cleavage activity of CRISPR/Cas12a, resulting in the cleavage of probe1, leading to the decrease of the electrochemical signal. The LOD of this biosensor was 10 pg/ml and the linear detection range was from 100 to 50000 pg/ml (Chen et al., 2022). Moreover, aptamer technology has also been implemented in the detection of other cardiac biomarkers, such as B-type natriuretic peptide (Tu et al., 2020), C-reactive protein (Wang et al., 2017), and myoglobin (Nia and Azadbakht, 2018).

Lipid abnormalities are another major risk factor for cardiovascular events, and the plasma lipid panel, consisting of triglycerides, low-density lipoprotein cholesterol (LDL-C), high-density lipoprotein cholesterol (HDL-C), and total cholesterol, is associated with CVD risk. By leveraging the versatility of aptamers, ligands that bind to LDL particles have been successfully developed, demonstrating clinically relevant diagnostic assays. An encoder system, consisting of bare gold nanoparticles (AuNPs), AuNPs-anti-LDL aptamer, and AuNPs-non-aptamer DNA, has been introduced to obtain entire information for serum lipoprotein subclasses, as well as accurately identified LDL at 0.05–37.5 ug/mL. A DNA origami-based aptamer nanoarray has also been generated, which can specifically bind to thrombin molecules to elicit a potent anticoagulant activity (Zhao et al., 2021). Fluorescent upconverting nanoparticles (UCNPs) with the size of 100 nm were applied as a conjugated form with the aptamer in a lateral flow biosensor. Sandwich-type hybridization models were performed in that biosensor had a pair of cognate aptamers to dual bind with visceral adipose tissue-derived serine protease inhibitor vaspin, one (capture probe) is coated on the test zone and the other (detection probe) is labeled by UCNPs. This aptasensor can detect vaspin concentration in a linear range from 0.1 ng/ml to 55 ng/ml, while the LOD was down to 39 pg/ml (Ali et al., 2020). These nanostructure-based aptasensors with ultrasensitive and high reproducible capability, provide an excellent determination approach for cardiac disease diagnosis in near future.

Besides, testing thrombosis is also important for the diagnosis of lesions, fibrin is a crucial molecule constituent of blood clots, and can be utilized in lesion imaging. Fibrinogen aptamer (FA), the blood clot-seeking moiety, conjugates to Gd (III)-NOTA (NOTA = 1,4,7-triazacyclononane-1,4,7-triacetin acid), chelates as contrast agent, measured by magnetic resonance imaging (MRI), significantly improving the contrast capabilities toward molecular imaging of thrombi (Koudrina et al., 2021). In another study, FA was incorporated with AuNPs, showing superior concentration-dependent contrast enhancement capabilities in computed tomography and fluoroscopy imaging (Koudrina et al., 2020). Early interrogation of disease is important for treatment and compared to the existing techniques for in medicinal centers, aptamer-based sensors tend to be higher in sensitivity and selectivity in the academic laboratory. However, rarely clinical practice of aptasensor has been underway, regarding to the lack of high-impact aptamers, expensive synthesis cost, and complex sample matrix. To date, more effort is needed to shift laboratory research to clinical applications in the future.

## Aptamer-functionalized therapeutic strategy

Atherosclerosis is a chronic inflammatory disease that originally starts from endothelial injury, followed by lipid deposition, impaired resolution of persistent inflammation, as well as thrombosis. Currently, lipid-lowering statins and anti-inflammatory agents are the cornerstones of atherosclerosis therapy to stabilize and reduce plaques (Xu et al., 2021). However, residual cardiovascular risk in certain groups of patients remains very high and some patients have poor responsiveness to statins. Thus, an additional new class of anti-atherosclerosis drugs is needed (Xu et al., 2018). As mentioned above, the aptamer is an alternative biorecognition tool for judging cardiac disease. They also have strong potential in drug exploitation and targeted therapy (Cen et al., 2021).

### Aptamers as therapeutic agents

Pegaptanib sodium (Macugen), which prevents angiogenesis, has gained approval by the US Food and Drug Administration (FDA) as an anti-vascular epidermal growth factor RNA aptamer for a patient with age-related macular degeneration (Cen et al., 2021). This approval presents the potential that aptamers can be developed as therapeutic agents.

Immediate Percutaneous Coronary Intervention (PCI) within 2 hours is significant for acute coronary syndromes (ACS) patients to reduce mortality, particularly those with ST-segment elevation MI (STEMI) (Bhatt et al., 2022). In recent guidelines, the drug-eluting stent (DES) is recommended over bare-metal stents (BMS) (Class of Recommendation I, Level of Evidence: A) for any PCI irrespective of clinical presentation (Collet et al., 2021), and new-generation DES with more biodegradable polymers were developed to improve the biocompatibility of stents (Chichareon et al., 2019). Most of the eluting drugs commonly used are antitumor drugs (rapamycin and paclitaxel) suppressing the proliferation of vascular smooth muscle cells while inevitably restraining the repair of vascular endothelial cells, thus causing a decrease in endothelial cell coverage of the stented segment and impairment of vascular endothelial function (Torii et al., 2020). Although studies show that DES lowers the incidence of in-stent restenosis (ISR), it also leads to secondary pathological changes in the stented vessel segment, such as endothelial cell inclusion diminution, platelet aggregation, and inflammatory cell infiltration at stenting sites, even triggering the development of neoatherosclerosis and elevated risk of intrastent thrombosis (Kerkmeijer et al., 2018; Ben-Yehuda, 2020). Therefore, postoperative dual antiplatelet therapy (DAPT) is a basis for the management of patients after DES implantation to reduce the formation of blood clotting, while raising the risk of bleeding. Bleeding, as a common complication after PCI, impacts the short- and long-term survival rates of patients with CAD (Yin et al., 2019).

Von Willebrand Factor (vWF), a giant multimeric glycoprotein, serves as a bridge between exposed collagen and platelet glycoprotein GPIb, enabling platelets to tether to vascular injury sites and initiating the formation of thrombus, which may lead to occlusive thrombi in coronary and cerebral arteries (Zhu et al., 2020a; Zhu et al., 2020b). It is released from endothelial cells and platelets at sites of vessel damage (Siller-Matula et al., 2012). Conventional treatment of ischemic disorders involves antiplatelet therapy, while mostly focusing on platelet activation and aggregation other than vWF activity. As vWF is a major contributor to atherogenesis in the arterial circulation, it can be identified as an appealing novel target for the intervention of cardiovascular diseases. Anti-vWF aptamers have been developed to block the GPIb-vWF axis and were initially confirmed by preclinical and clinical investigation. ARC1172 is a 41-mer DNA aptamer blocking the A1 domain of vWF to inhibit interaction with platelets GPIb(Li et al., 2014). ARC1779 was derived from ARC1172 by introducing modifications, such as inverting a phosphorothioate linkage between G21 and T22, conjugating 20kDA polyethylene glycol to the 5′-G residue, or depleting the terminal base pair from stem-loop. The inhibitory effect of ARC1779 on vWF activity and shear-dependent platelet function is dose- and concentration-dependent in healthy volunteers in phase 1 investigation (Gilbert et al., 2007), as well as increased vWF activity in MI *ex vivo*. In a randomized clinical trial, the results demonstrate that ARC1779 reduces cerebral thromboembolism in patients undergoing carotid endarterectomy (Markus et al., 2011). Notably, the research indicated that ARC1779 did not interfere with other platelet aggregation pathways at concentrations that fully suppressed vWF-mediated platelet activity with less bleeding compared to GPIIb/IIIa inhibitors. ARC15105 as second generation anti-vWF aptamer also shows a potent antagonistic effect on the aggregation of platelet induced by a variety of activators such as ristocetin, ADP, collagen, and arachidonic acid in samples from MI patients and healthy individuals. Compared with its’ predecessor ARC1779, ARC15015 has a longer half-life (6–8 h) and higher bioavailable with 2′OME modified chemistry (Siller-Matula et al., 2012). However, ARC15015 is not steady at a high temperature of 37 °C due to its short base pairs in a typical stem-loop structure. BT100 is generated with four more extra base-pairs added using phosphoramidite solid-phase chemistry and pegylated by conjugation chemistry to develop BT200. BT200, as a pegylated synthetic ssRNA, efficiently interdicts the vWF-GPIb axis, favoring the arterial over the venous circulatory system (Zhu et al., 2020a; Zhu et al., 2020b), and can be rapidly antagonized by specific reversal agents BT101 (Zhu et al., 2020b). A recent study showed that BT200 can also effectively lower high vWF levels in large artery atherosclerosis stroke patients and could be a prominent candidate for secondary stroke prevention (Kovacevic et al., 2021). In another research, DTRI-031, an optimized vWF aptamer, has been synthesized which can not only prevent plate-rich thrombosis in carotid artery injury models but also restore blood flow following arterial occlusion. Compared to alteplase, the only therapeutic agent approved by the FDA for arterial recanalization, DTRI-031 represents superior antithrombotic potency and reversibility (Nimjee et al., 2019). TAGX-0004 as a new generation of anti-vWF aptamer also had a superior affinity to the vWF A1 domain and could inhibit platelet aggregation *ex vivo*, potentially could be developed as an appealing agent not only for the treatment of acute Thrombotic Thrombocytopenic Purpura but also arterial thrombotic disorders (Matsumoto and Harada, 2022). VWF is now becoming recognized as a mediator of atherosclerotic disorders due to its casual effect on shear-dependent thrombosis formation and can be an indicator of the presence and prognosis of cardiovascular events. Targeted inhibition of vWF represents a distinct opportunity to improve the therapeutic armamentarium for the management of ACS and PCI.

Thrombus formation often causes major cardiovascular events and results from two primary pathways, involving coagulation proteins and platelets. Conventional drugs for inhibiting thrombosis include antiplatelet drugs (aspirin, abciximab, and tirofiban) and anticoagulants (heparin, warfarin, and apixaban) (Steffel et al., 2018; Zhu et al., 2020a). Thrombin is a vital enzyme in the coagulation cascade, as well as an attractive target for the design of anticoagulant drugs. A series of thrombin-binding aptamers have been screened by SELEX, which can directly recognize and inhibit thrombin (Lai et al., 2018; Derszniak et al., 2019; Bao et al., 2021). RNA aptamers as reversible antagonists were first introduced in 2002, Rusconi et al. selected the oligonucleotides against FIXa and designed antidotes that could effectively inactivate the anticoagulant inherent of aptamers. With their poor immunogenicity, aptamers offer alternatives for patients developing heparin-induced thrombocytopenia, an immunological response that is not authorized with heparin for further administration and is superior to antibodies Rusconi et al. (2002). Pegnivacogin, a novel RNA-aptamer-based FIXa inhibitor, features platelet activity reduction in acute coronary syndromes (ACS) patients (Staudacher et al., 2019). Plasma kallikrein is a serine protease in coagulation, which amplified the generation of activated factor XIIa (FXIIa), ultimately leading to thrombin generation and fibrin clot formation. A kallikrein-targeting RNA aptamer has been isolated that can inhibit the intrinsic pathway of coagulation and reduce bradykinin release, providing an alternative to traditional anticoagulants with anti-inflammatory properties (Steen Burrell et al., 2017). Thrombin binding aptamer (TBA) is an artificial DNA sequence to inhibit fibrin-clot formation. Bao et al. succeeded in the synthesis of 8-trifluoromethyl-2′-deoxyguanosine (^F^G) and incorporated it into the TBA sequence, enhancing the pharmacodynamic stability and antithrombosis activity of TBA (Bao et al., 2021). Administrations of aptamers to against thrombosis are under clinical trials. ARC183 (Bock et al., 1992), a DNA aptamer selected against thrombin, has passed the Phase I trial. Another anti-coagulant aptamer is RB006, targeting against FIXa, which has been also entered into clinical trials with its complementary antidote RB007 as an anticoagulant system (REG1, Regado Biosciences) (Gopinath, 2008).

Endothelial dysfunction is also regarded as a hallmark of multiple aspects of panvascular disease, being the driving force for stroke, hypertension, diabetes, and worsening renal function. Dysfunction of the vascular endothelium mainly manifests as aberrant cell metabolism, oxidative stress, immoderate inflammatory response, and endothelial-to-mesenchymal transition (Xu et al., 2021). Nitric oxide (NO) derived from endothelial cells is a key molecule in vascular homeostasis and its excess production may lead to endothelial dysfunction and pro-atherogenic events in the artery (Hong et al., 2019). The aptamer, designed for Selectin P Ligand Protein, exerted significant NO inhibition in atherogenic events through the moieties such as AKT serine/threonine kinase 1, estrogen receptor 1, and nitric oxide synthase-3. With the development of Cell-SELEX, blood-brain (BB) permeable aptamers have been identified *in vivo*. R39 (39 nt) aptamer was selected against immortalized endothelial cells *via* Cell-SELEX. It can recognize primary endothelial cells and be internalized *via* endocytosis, as well as carrying siVEGFR2 in a long polymeric form to efficiently reduce target gene expression (Dua et al., 2018). Integrin avβ3, expressed in endothelial cells, plays a critical role in the progression of vascular remodeling and atherosclerosis, and thereby avβ3 aptamer also exerts its inhibitory effect on vascular smooth muscle cell proliferation and migration *via* Ras-PI3K/MAPK signaling pathway, a promising therapeutic agent to treat post-PTCA vascular restenosis (Wu et al., 2020).

Furthermore, anti-proprotein convertase subtilisin/kexin9(PCSK9) aptamers have been generated using CE-SELEX, presenting efficient PCSK9 inhibition, which may be potential therapeutic agents for patients with hypercholesterolemia and CAD (Sattari et al., 2020). Nevertheless, conventional anti-thrombosis agents aggravate the risk of bleeding and there are few available antagonists (Nimjee et al., 2019). It is worth noting that ssDNA aptamers targeting Dabigatran Etexilate, an anticoagulant drug with a small molecular weight, have been generated with dissociation constants (K_d_) ranging from 46.8 to 208nM, which can be a prominent option for detecting Dabigatran action, as well as controlling over the risk of bleeding after drug therapy (Aljohani et al., 2018). Even though with encouraging preclinical trials, aptamer represents a promising approach to meet the medical need with an improved risk/benefit profile for the management of thrombotic events in major organs, still, the structural information of aptamers needs to be further explored to find suitable codes that can be more specific to target proteins. Likewise, their safety requires more evaluation due to the lack of sufficient clinical evidence.

### Aptamers as ligands for delivery

**FIGURE 3:**
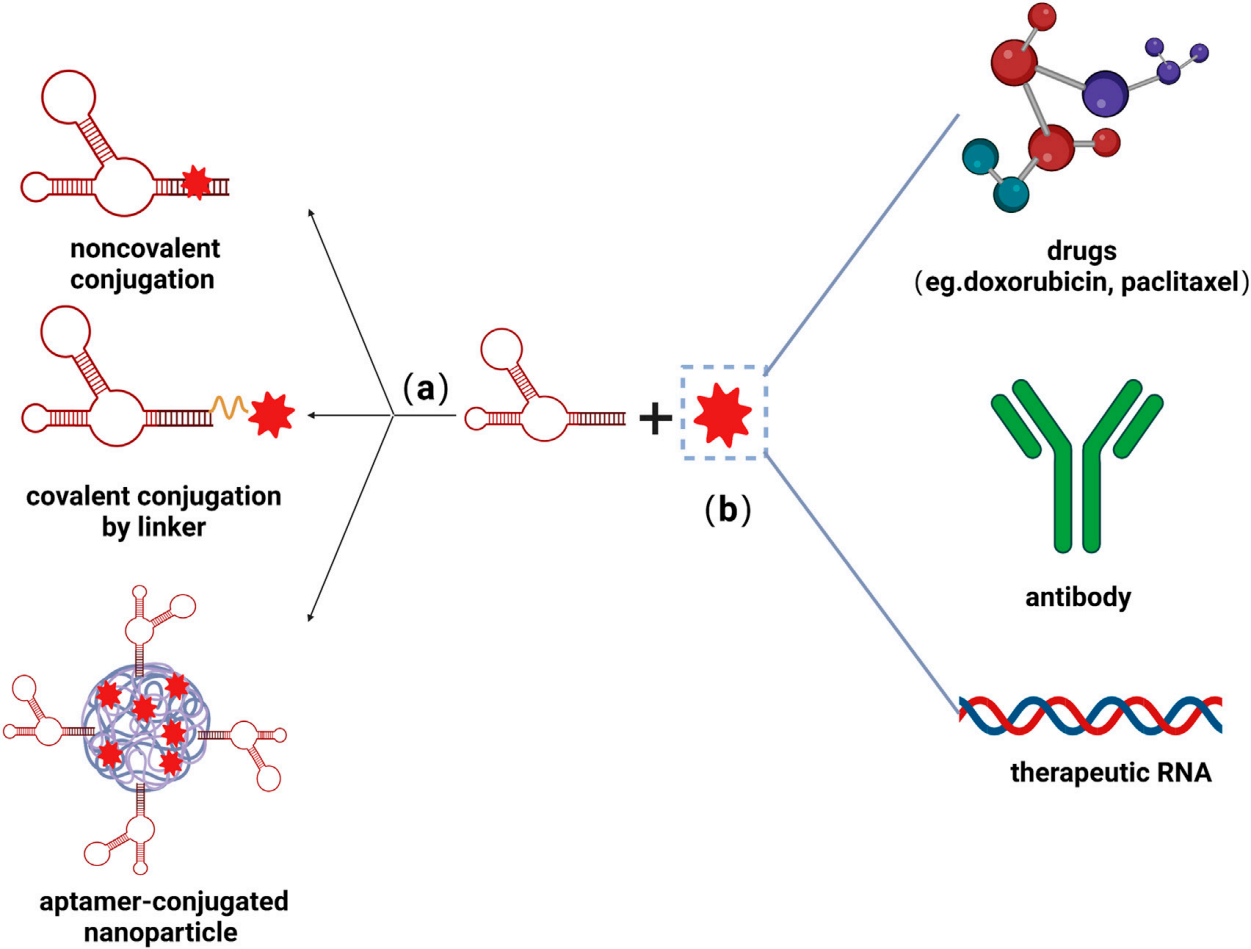
Schematic illustrations of aptamer-functionalized delivery system. **(A)** Nucleic acids can couple with small molecules *via* noncovalent conjugation, covalent conjugation by linker, as well as co-conjugate on the surface of nanoparticles. **(B)** Aptamers could be conjugate with various therapeutic molecules, including drugs, antibodies, and RNAs, greatly improving the efficacy of treatment. Created with BioRender.com.

In addition to applying as stand-alone therapeutics, aptamers have also been developed as the vehicles for targeted delivery tools conjugated to oligonucleotide, drug, and nanoparticles, as shown in Figure 3. Aptamers as escorts were originally introduced by Hicke et al., in 2000 Hicke and Stephens (2000), and afterward have been wildly studied in cancer therapy, including chemotherapy and immunotherapy (Zhu and Chen, 2018), while little research carried out on cardiovascular disorders. The delivery of curative agents through the circulatory system is one of the least invasive interventions and has garnered a great deal of attention in the cardiovascular field, yet this strategy is hampered by low vascular density or permeability (Huang et al., 2021). Recruitment of immune cells, such as splenic monocytes, takes place in the microenvironment to maintain physiological homeostasis (Mentkowski et al., 2020). Inspired by this phenomenon, several studies have reported cell-carrying nanoparticles as active cardiac targeting approaches. Lipid nanoparticle (LNPs) carrying IOX2, hypoxia-inducible factor (HIF)–1α prolyl hydroxylase–2 inhibitors, are able to attach onto the surface of monocytes and released to the damaged sites, ameliorating cardiac ischemia-reperfusion (IR) injury (Huang et al., 2021). Rusconi et al. Rusconi et al. (2016) generated a mimetic peptide (MP), which imitates an amino acidic stretch of the C-terminal tail of the Ca_v_β2 cytosolic chaperone, which can specifically target the Tail Interacting Domain (TID) within the Ca_v_β2 globular domain while facilitating the restoration of Ca_v_α1.2 protein density at the plasma membrane in heart conditions associated with altered L-type calcium channel (LTCC) density. However, how to deliver MP specifically to cardiac cell have not been elucidated. In a novel aptamer-peptide chimera, MP was conjugated with Gint4. T through Cu (I)-mediated click chemistry (Romanelli et al., 2018). Gint4. T is a nuclease-resistant ssRNA with translational potential, targeting platelet-derived growth factor receptor-β (PDGFRβ) and hence inhibiting cellular proliferation, migration, and angiogenesis (Camorani et al., 2014). The developed aptamer-peptide conjugates facilitate cell internalization of the MP to cardiomyocytes, thus showing its recovery effect in the cardiac context with abnormal LTCC-dependent intracellular calcium fluxes (Romanelli et al., 2018). The sarcoplasmic reticulum Ca^2+^-ATPase 2a (SERCA2a)-phospholamban (PLN) system plays a pivotal role to regulate the cycling of intracellular Ca^2+^ in ventricular cardiomyocytes. PLN-specific aptamer with a phosphorothioate-modified backbone has been isolated from a random library of an RNA sequence by SELEX and shortened to a 30-nucleotide oligomer, named RNA-Apt30. Conjugation of RNA-Apt30 to the cell-penetrating TAT peptide, through a stable thiol-maleimide linkage, allows its delivery into adult rat cardiomyocytes, in which it enhanced both cardiac Ca^2+^ transients and contractility (Sakai et al., 2014). Aptamers with the ability to capture endothelial progenitor cells (EPCs) were reported and assembled on the surface of the stents, showing great efficacy of re-endothelialization, which improves the hemocompatibility of the grafts (Hoffmann et al., 2008). Additionally, heparin, as an anticoagulant agent, was designed to fix onto the surface of the dopamine/polyethyleneimine film which was also immobilized with EPCs-capturing aptamers *via* electrostatic interaction, greatly improving the hemocompatibility of vascular stents (Deng et al., 2017).

In the field of cancer therapy, an aptamer-functionalized drug delivery system can achieve the enrichment of therapeutic agents in targeted cells, so as to promote the demise of neoplasm cells and impede the progression of tumor tissues. The reason that aptamer-drug conjugates (ApDCs) tend to be less studied in cardiac therapy may be that cardiomyocytes are non-regenerative in nature, so the attacks mainly concentrate on antiangiogenic and antithrombotic regimens. Perhaps afterward, we can take up a series of studies on those cells which are abnormally hyperactivated in the pathogenesis of MI. Recently, increasing observations have shown that the pathobiology of atherosclerosis is closely related to the dysfunction of cellular immunity (Gaddis et al., 2019) and highlights potential T cell-related immunotherapies in cardiovascular disease (Saigusa et al., 2020). Notably, with the advancement of gene engineering, aptamers have been reported that serve as cargo to specifically guide therapeutic RNA to target cells, such as small interference RNA (siRNA). The emergence of CD4 aptamers, which specifically bind to the surface of CD4^+^ T cells and transport small molecules into them, allowing it as T helper (Th) cell-specific delivering carriers. CD4 aptamer-RORγt siRNA chimeras have been developed to inhibit pathogenic effector functions of Th17 cells. Besides, CD4 aptamer bearing RORγt-short hairpin RNA (shRNA) also manipulates that decreased IL-17 release from Th cells and suppressed Th17 cell differentiation (Lin et al., 2015). Th17 cells play a vital role in promoting the inflammatory response during cardiac remodeling (Sun et al., 2021) and the development of CD4 aptamers probably be novel tools in the treatment of atherosclerotic disorders, bringing precise therapeutic perspectives. Along with the emerging progression of multiple compound targeted drug delivery systems, aptamer technology has shown clinical treatment possibility in the field of panvascular disease treatment.

## Conclusion and future perspectives

**FIGURE 4:**
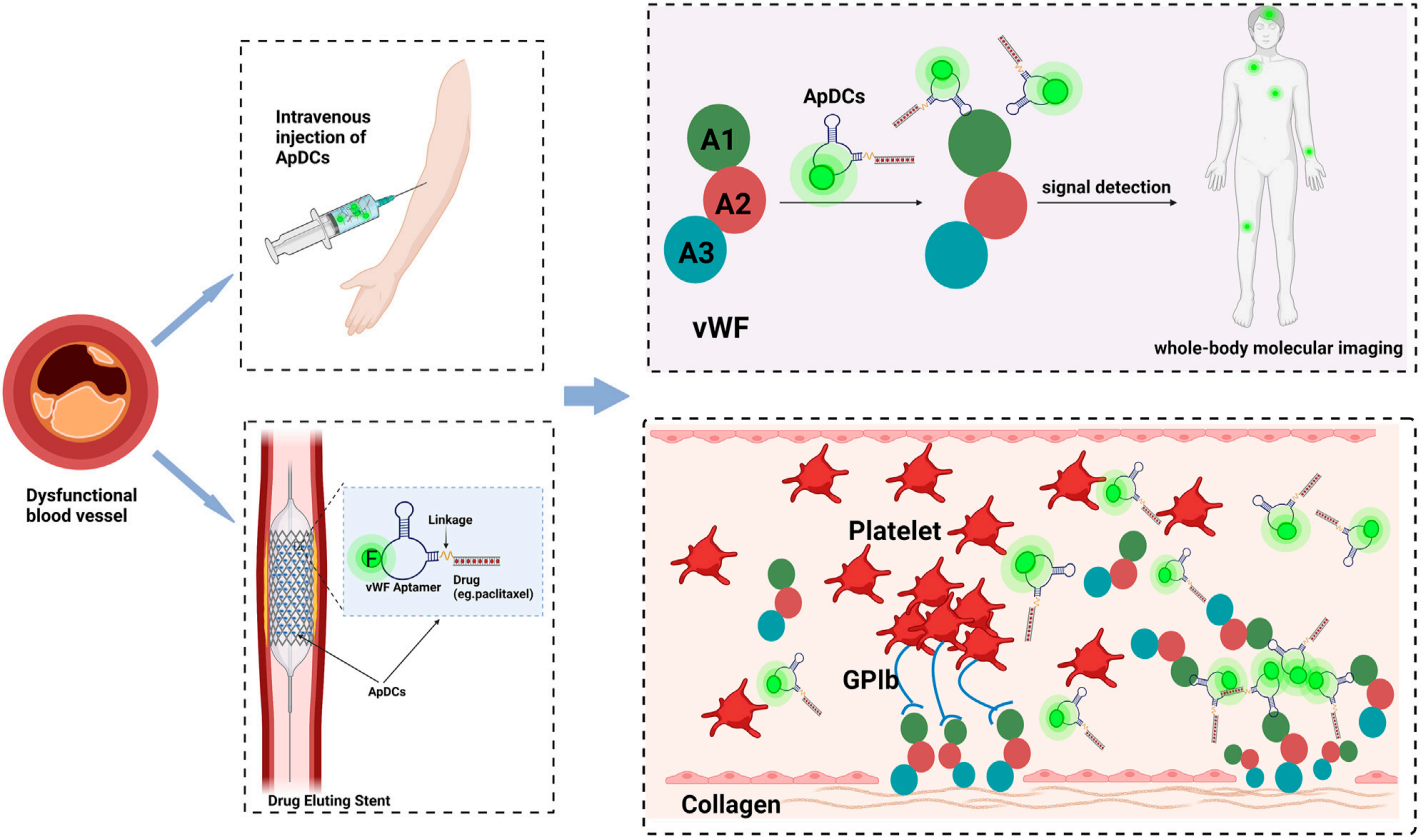
Aptamer-based applications in cardiovascular disease to realize the integration of diagnosis and treatment in the future. (Take vWF as an instance.). The drug is coupled to the vWF aptamer by covalent bonding to construct the aptamer-drug conjugates and then injected intravenously or subcutaneously. Aptamer-drug conjugates can be also immobilized on the surface of the stent, and enters the body with the stent implantation. As they will assemble at the sites of stressed endothelium, the relevant signals at lesions can be captured by detection machines, meanwhile lifting the drug concentration at the injured sites locally, to establish a point-to-point interface to achieve controlled drug release, reducing the complications of bleeding and realizing disease treatment with visualization. Created with BioRender.com.

The past decade has witnessed biomedical technology accelerating towards a new era of precision medicine for human health. As etiological mechanisms of cardiovascular diseases continue to advance, especially the understanding of panvascular conditions, the spectrum of overall vascular disease has been formed, while there are certain limitations in diagnosis and treatment. Currently, techniques such as stent implantation or balloon dilatation have been wildly used in the cure of cardiovascular diseases, followed by in-stent thrombosis and bleeding complications in the organism. Accompanied by burgeoning interdisciplinary approaches, nucleic acid aptamers present great potential for clinical practice, due to their unique attributes, including specificity, high binding affinity, ease of cellular internalization, and rapid tissue accumulation capabilities, significantly improving the sensitivity and accuracy of early atherosclerosis diagnosis. The usage of aptamers combined with drugs or small molecules can also complement diagnostic information and share synergistic advantages, breaking the boundaries of single diagnostic imaging, and optimizing comprehensive management strategies for cardiovascular diseases. As shown in Figure 4, vWF aptamers have been developed to inhibit thrombosis and afterward, we could proceed to label them with tags and map them with relevant detection tools, thus enabling the imaging of the specific vessels where thrombosis occurs, while simultaneously administering antithrombotic therapy, thus realizing targeted therapy in visualized situations. On the other hand, we could set about conjugating vWF aptamers with agents (rapamycin, paclitaxel, zotamox, *etc.*) to construct ApDCs, which can be co-assembled onto the surface of stents or balloons to prevent the occurrence of in-stent regenerated clots by releasing drugs specifically at the stent restenosis site, exploiting the characteristic of increased vWF production at the location of stressed endothelial cells. What’s more, this model has a tissue feedback-friendly interface with controlled drug release, which could change the current management pattern of oral DAPT after PCI, thus greatly reducing or avoiding the occurrence of corresponding complications such as bleeding, thereby further promoting the long-term prognosis.

Of note, numerous pieces of research have been conducted on the development of aptamers, while there are also some limitations and few aptamers have successfully entered clinical trials. Further efforts should be made to overcome their drawbacks and bring them into cardiovascular precision medicine: 1) Some fundamental details about aptamers should be further explored to better their performance, such as their structures, binding affinities, and regulation of protein or gene expression. 2) Applying more reliable methods for the selection, synthesis, and modification of aptamers for practical applications. 3) Aptamers as therapeutic agents need to be developed and transformed into medical utility to rapidly improve diagnostic and intervention processes.
